# Effects of childcare disagreement with children, social support, and health status on unmet healthcare-seeking behavior among the migrant older with children to Jinan, China

**DOI:** 10.3389/fpubh.2022.957619

**Published:** 2022-10-10

**Authors:** Xinfei Shi, Di Zong, Zhongqian Lu, Shixue Li, Fanlei Kong

**Affiliations:** ^1^Centre for Health Management and Policy Research, School of Public Health, Shandong University, Jinan, China; ^2^NHC Key Lab of Health Economics and Policy Research, Shandong University, Jinan, China

**Keywords:** unmet healthcare-seeking behavior, childcare disagreement with children, social support, health status, migrant older with children

## Abstract

Due to the acceleration of China's urbanization, the number of migrant older with children (MOC) continued to increase. This study aimed to clarify the effects of childcare disagreement with children, social support, and health status on unmet healthcare-seeking behavior among the MOC to Jinan, China. A cross-sectional study included 656 MOC (36.3% men and 63.7% women) using multi-stage cluster random sampling in Jinan, China. Childcare disagreement was evaluated by the differences between parents and grandparents on the diet, dressing, education, and childcare consumption. Social support was assessed using the social support rating scale (SSRS). Descriptive analysis, chi-squared test, and binary logistic regression were applied to analyze the association between childcare disagreement with children, social support, health status, and unmet healthcare-seeking behavior of the MOC. Approximately 41.3% of participants had unmet healthcare-seeking behavior. Logistic regression analysis showed that the MOC whose health status compared to last year get better were more likely to have unmet healthcare-seeking behavior, while who were women, had partial will of migration, hired a nanny, had smaller childcare disagreement with children on dressing, had smaller childcare disagreement with children on consumption, and had moderate social support, were less likely to experience unmet healthcare-seeking behavior. Recommendations were given to the government and family members to improve the health services-seeking behavior of the MOC.

## Introduction

With the fast population aging in recent decades, China owned the largest number of older adults in the world (1). According to the latest data from the China National Bureau of Statistics, older adults aged 60 and more was 264 million in 2020, accounting for 18.7% of the total population of China. Of these, population aged 65 and older was 190 million, accounting for 13.50% of the total population of China. Compared with 2010, the proportion of people aged 60 and over has increased by 5.44%, and the proportion of people aged 65 and over has increased by 4.63% (2). Migrants were defined as individuals who have resided in the destination place for at least 6 months without local household registration status in China (3). In 2015, China's migrant population reached 247 million, accounting for 18% of the total population, and the trend of household mobility was increasing. According to the National Health and Family Planning Commission, the number of older migrants accounted for 7.2% of the total migrants in 2015. Of them, 43% migrated to take care of the younger generation (4). However, the disconnection of medical insurance between the inflow city and their hometown, unfamiliarity with the local medical environment, different rights to use healthcare services, etc. put the health of the migrant older with children (MOC) at risk. Maintaining and promoting the population health and providing healthcare services had always been the concern of the government (5). Thus, clarifying unmet healthcare behavior and needs of the MOC became very important for the government and healthcare service researchers.

Unmet healthcare needs are defined as a state in which individuals do not have access to healthcare when needed (6, 7). Healthcare-seeking behavior, defined as a “tool for investigating the individual's or population's interaction with the health system,” is fit-for-purpose to study how people perceive health and how they access and use available services to promote it (8). Insufficient opportunities to seek healthcare may lead to inappropriate or delayed healthcare, leading to adverse outcomes, such as a high prevalence of infectious diseases (9). Many studies reported the poor healthcare-seeking behavior, for example, in South Korea, and more than 14.5% of research subjects reported unmet healthcare needs since 2009 (10). Based on qualitative data from in-depth interviews with 90 rural-to-urban migrants, Hong's study found that migrants had limited access to regular medical services (11). Xie's study found that aged rural migrant workers who moved to the east or to first- or second-tier cities were less likely to seek medical care (12).

The older migrant who had disagreement with children may affect their healthcare-seeking behavior. A previous study showed that the living habits and consumption concept of the MOC were quite different from their children, and that they were easy to have friction with their children (13). A study of older immigrants in the USA found that those with more negative family relations had more doctor visits and were marginally more likely to use inpatient services, indicating that family conflict was a predictor of healthcare service use (14). Family conflict was also shown to be associated with mental health status and the use of healthcare services among Chinese Americans (15).

Social support refers to communication and contact between individuals and others for the purpose of obtaining information and comfort (16). The effect of social support on health has been widely reported. A study of Japanese older adults showed that respondents who received instrumental support were less likely to experience unmet healthcare needs, except for men aged 65–69 (17). In the USA, social support for older people has been shown to delay further deterioration in their health (18). Some scholars pointed out that social support was positively related to the health status of older people in developing countries (19). It was also found that medical decisions made by the older migrant was associated with their family support in China (20). Existing research also found that social support did not promote the physical health of older people in rural areas in China, but had significant positive effects on their mental health, especially in terms of emotional support (21).

Studies on the relationship between health status and unmet healthcare-seeking behavior mainly indicated that health status was an important factor affecting the healthcare-seeking behavior of migrants, yet limited studies focused on the MOC. It was found that the healthcare-seeking behavior of the older migrant in China were affected by self-rated health, income, employment status, and medical insurance participation no matter they were from rural to urban area or from urban to urban area (5). A study showed ~70.7% of the older migrant used inpatient services when they suffered from diseases requiring hospitalization (22). Residents of difficult-to-reach areas had a high prevalence of health problems and experienced social and structural barriers to healthcare services access (23). Patients with good self-rated health status were more likely to have an unmet need for outpatient services compared with those with poor health status (24).

To conclude, no study simultaneously explored the association between childcare disagreement with children, social support, health status, and unmet healthcare-seeking behavior, let alone took MOC as the object of research. Thus, this study aimed to clarify the effects of childcare disagreement with children, social support, health status on unmet healthcare-seeking behavior among the MOC in Jinan, China, and to further provide evidence-based suggestion for China and other developing countries facing similar challenges.

## Methods

### Data source

The data were collected in August 2020 in Jinan, Shandong province, China. Shandong province is located in eastern China, and Jinan City is the provincial capital of Shandong province. In 2020, its GDP will be 1.01 trillion Chinese Yuan (CNY) (≈157,285.51 million USD) (25). As of 1 July 2020, Jinan has jurisdiction over 10 districts and two counties (132 subdistricts and 29 towns) (26). By the end of 2020, the local resident population was 9.20 million, while the registered population was 8.06 million (27). In 2020, the migrants in Jinan were 3.29 million (28). Migrants aged above 60 and followed their children to Jinan City were the participants of this study.

Multi-stage cluster sampling was employed to select study participants. In the first stage of data collection, three districts were chosen from the 10 districts as the primary sampling units (PSUs), considering their economic development and geographic location. In the second stage, a total of three sub-districts were selected from each of the PSUs as the secondary sampling units (SSUs). In the third stage, three communities were selected from each of the SSUs. All older migrants who were over 60 years and followed their children to Jinan in these three communities constituted the total sample of this study.

A total of 33 college students became study investigators after training on study background information, questionnaire content, and social survey techniques. Of these students, 11 investigators came from Shandong University, 13 from Jinan University, two from Dongying Vocational College, and seven from Weifang Medical University. Participants were conducted face-to-face interviews for ~20 min to collect the data. Initially selected and 670 MOC were interviewed. However, 14 of them were excluded due to obvious logical errors in their answer or an unfinished questionnaire. A total of 656 participants eventually participated in the study.

### Variables

#### Dependent variable

The dependent variable for the study was defined by asking respondents two questions, “Did you have the experience of not taking the outpatient service in case of disturbing your child?” and “Did you have the experience of not taking the inpatient service in case of disturbing your child?”, and it was calculated by combining these two variables to a new variable named unmet healthcare-seeking behavior. Unmet healthcare-seeking behavior had two options, “yes” and “no.” The “yes” refers to having at least one experience and “no” means the absence of these two experiences.

#### Independent variables

##### Demographic characteristics

Demographic characteristics included gender, age, body mass index (BMI), source of living expenses, marriage, employment, education level, monthly income, household monthly income, Hukou (commonly known as household registration, every person is assigned a type of Hukou based on his or her birthplace), will of migration, and hired a nanny. Participants' age is described by the mean and standard deviation (SD); education level including middle school and below, high school, and high school and above; and marriage status (married, unmarried, divorced, widowed, and others). BMI was calculated as weight divided by height squared (kg/m^2^). A BMI < 18.5 kg/m^2^ was considered underweight, between 18.5 and 23.9 kg/m^2^ was considered normal, between 24 and 27.9 kg/m^2^ was considered overweight, and ≥28 kg/m^2^ was considered obese (29). There were four types of migration space range: trans-county, trans-city, trans-provincial, and trans-national.

##### Childcare disagreement with children

Childcare disagreement was assessed by the childcare differences between parents and grandparents on diet, dressing, education, and consumption. Childcare disagreement with children was captured by four indicators, including “differences with childcare disagreement with children on diet (30),” “childcare disagreement with children on dressing,” “childcare disagreement with children on education (31),” and “childcare disagreement with children on consumption (13).” All of these variables had four options, “none,” “smaller,” “larger,” and “completely.”

##### Social support

Social support was assessed using the social support rating scale (SSRS), which was developed by Xiao Shuiyuan in 1994 for the Chinese population. It is composed of three dimensions: subjective support, objective support, and support utilization (32), which contains 10 kinds of support: friends, residents, neighbors, colleagues, family members, economy, comfort, talk, help, and activities. The higher the total social support score, the more social support the subjects received. The full score is 66 points; a total score of ≤22 points is the low level; 23 ≤ total score ≤44 is the medium level; 45 ≤ total score ≤66 is considered to be the high level (33).

##### Health status

Health status included “health status compared to peers (34)” and “health status compared to last year (35).” Respondents were asked about the variable of health status compared to peers “How is your health like compared to your peers?” Respondents were asked about the variable of health status compared to last year “How is your health like compared to last year?”

### Statistical analysis

All data were analyzed with SPSS 26.0, and *p* < 0.05 was considered statistically significant. The percentages of the nominal variables were determined. Firstly, a descriptive analysis and the chi-squared test were performed to show the relationship between related variables and unmet healthcare-seeking behavior. Four binary logistic regression models were then adopted to clarify the associations between childcare disagreement with children, social support, health status, and unmet healthcare-seeking behavior. Meanwhile, crude odds ratios (ORs) and 95% confidence intervals (95% CI) were calculated. In Model 1, basic demographic information variables were included; Model 2 included basic demographic information and childcare disagreement with children; Model 3 included demographic, childcare disagreement with children, and social support; and Model 4 included demographic, childcare disagreement with children, social support, and health status.

## Results

### Demographic characteristics

Table 1 shows the basic demographic information of the 656 MOC in Jinan, China. Overall, 41.3% of participants had unmet healthcare-seeking behavior. Approximately 36.3% of participants were men and 63.7% were women. Almost half of participants had source of living expenses of child support (49.7%). The majority of the MOC had a monthly income level of CNY0–100 (USD0–15.5) (33.7%) and a household monthly income level of CNY101–600 (USD15.6–92.7) (29.6%). More than half of participants were willing to migrate (67.7%). Most participants had no nanny (99.1%). A statistically significant difference between gender, source of living expenses, monthly income, household monthly income, will of migration, hired a nanny, and unmet healthcare-seeking behavior were found based on the chi-squared test among the MOC in Jinan, China.

**Table 1:** Demographic characteristics of the migrant older with children (MOC).

**Variables**		**Unmet healthcare-seeking behavior**			***χ* ^2^**	***P***
		**Total *n* (%)**	**No *n* (%)**	**Yes *n* (%)**		
**Observations**		656 (100.0)	385 (58.7)	271 (41.3)		
**Gender**					7.244	0.007
	Male	238 (36.3)	156 (40.5)	82 (30.3)		
	Female	418 (63.7)	229 (59.5)	189 (69.7)		
**Age**					0.407	0.939
	60–62	126 (19.2)	74 (19.2)	52 (19.2)		
	63–65	197 (30.0)	117 (30.4)	80 (29.5)		
	66–68	183 (27.9)	104 (27.0)	79 (29.2)		
	≥69	150 (22.9)	90 (23.4)	60 (22.1)		
**BMI**					4.045	0.257
	≤18.4	23 (3.5)	14 (3.6)	9 (3.3)		
	18.5–23.9	259 (39.5)	141 (36.6)	118 (43.5)		
	24–27.9	300 (45.7)	188 (48.8)	112 (41.3)		
	≥28	74 (11.3)	42 (10.9)	32 (11.8)		
**Source of living expenses**					10.999	0.027
	Pension or salary	194 (29.6)	107 (27.8)	87 (32.1)		
	Savings	41 (6.3)	21 (5.5)	20 (7.4)		
	Spouse's income	60 (9.1)	44 (11.4)	16 (5.9)		
	Child support	326 (49.7)	187 (48.6)	139 (51.3)		
	Basic living allowances	35 (5.3)	26 (6.8)	9 (3.3)		
**Marital situation**					2.394	0.664
	Married	583 (88.9)	342 (88.8)	241 (88.9)		
	Unmarried	1 (0.2)	0 (0.0)	1 (0.4)		
	Divorced	5 (0.8)	4 (1.0)	1 (0.4)		
	Widowed	58 (8.8)	34 (8.8)	24 (8.9)		
	Others	9 (1.4)	5 (1.3)	4 (1.5)		
**Employment status**					1.250	0.535
	Employed	37 (5.6)	20 (5.2)	17 (6.3)		
	Retired	131 (20.0)	82 (21.3)	49 (18.1)		
	Unemployed	488 (74.4)	283 (73.5)	205 (75.6)		
**Educational level**					5.083	0.079
	Middle school and below	340 (51.8)	186 (48.3)	154 (56.8)		
	High school	192 (29.3)	118 (30.6)	74 (27.3)		
	Above high school	124 (18.9)	81 (21.0)	43 (15.9)		
**Monthly income**					16.396	0.001
	CNY 0–100 (USD 0–15.5)	221 (33.7)	142 (36.9)	79 (29.2)		
	CNY 101–600 (USD 15.6–92.7)	155 (23.6)	75 (19.5)	80 (29.5)		
	CNY 601–2,000 (USD 92.8–309.2)	177 (27.0)	96 (24.9)	81 (29.9)		
	CNY ≥ 2,001 (USD ≥ 309.2)	103 (15.7)	72 (18.7)	31 (11.4)		
**Household monthly income**					17.187	0.001
	CNY 0–100 (USD 0–15.5)	152 (23.2)	91 (23.6)	61 (22.5)		
	CNY 101–1,000 (USD 15.6–154.5)	194 (29.6)	99 (25.7)	95 (35.1)		
	CNY 1,001–2,500 (USD 154.7–386.3)	133 (20.3)	70 (18.2)	63 (23.2)		
	CNY ≥ 2,501 (USD≥386.4)	177 (27.0)	125 (32.5)	52 (19.2)		
**Hukou**					1.366	0.242
	Rural	574 (87.5)	332 (86.2)	242 (89.3)		
	Urban	82 (12.5)	53 (13.8)	29 (10.7)		
**Migration space range**					4.523	0.210
	Trans-county	146 (22.3)	80 (20.8)	66 (24.4)		
	Trans-city	441 (67.2)	267 (69.4)	174 (64.2)		
	Trans-provincial	66 (10.1)	35 (9.1)	31 (11.4)		
	Trans-national	3 (0.5)	3 (0.8)	0 (0.0)		
**Will of migration**					43.318	0.001
	Definitely not	14 (2.1)	8 (2.1)	6 (2.2)		
	Partially not	12 (1.8)	7 (1.8)	5 (1.8)		
	Generally	34 (5.2)	17 (4.4)	17 (6.3)		
	Partially will	152 (23.3)	56 (14.5)	96 (35.4)		
	Definitely will	444 (67.7)	297 (77.1)	147 (54.2)		
**Hire a nanny**					4.410	0.036
	No	650 (99.1)	384 (99.7)	266 (98.2)		
	Yes	6 (0.9)	1 (0.3)	5 (1.8)		

*MOC, migrant older with children; BMI, body mass index; CNY, Chinese Yuan, 1 CNY = 0.1545 USD; Trans-county refers to Chinese internal migrant flowing into the urban area from a county or village in Jinan; Trans-city refers to Chinese internal migrant flowing into Jinan from another city in Shandong province; Trans-provincial refers to Chinese internal migrant flowing into Jinan City of Shandong province from other provinces*.

### Childcare disagreement with children

Table 2 shows childcare disagreement with children of the MOC. Most of the MOC had no childcare disagreement with children on diet (76.1%), 78.5% had no childcare disagreement with children on dressing, and most people had no childcare disagreement with children on education (79.7%). Approximately 72.4% of participants never had childcare disagreement with children on consumption. A statistically significant difference between childcare disagreement with children on diet, childcare disagreement with children on dressing, childcare disagreement with children on education, childcare disagreement with children on consumption, and unmet healthcare-seeking behavior was found based on the chi-squared test among the MOC in Jinan, China.

**Table 2:** Childcare disagreement with children of the MOC.

**Variables**		**Unmet healthcare-seeking behavior**			***χ^2^***	***P***
		**Total *n* (%)**	**No *n* (%)**	**Yes *n* (%)**		
**Childcare disagreement with children on diet**					98.776	0.001
	None	499 (76.1)	346 (89.9)	153 (56.5)		
	Smaller	124 (18.9)	29 (7.5)	95 (35.1)		
	Larger	4 (0.6)	2 (0.5)	2 (0.7)		
	Completely	29 (4.4)	8 (2.1)	21 (7.7)		
**Childcare disagreement with children on dressing**					101.936	0.001
	None	515 (78.5)	354 (91.9)	161 (59.4)		
	Smaller	103 (15.7)	19 (4.9)	84 (31.0)		
	Larger	5 (0.8)	2 (0.5)	3 (1.1)		
	Completely	33 (5.0)	10 (2.6)	23 (8.5)		
**Childcare disagreement with children on education**					89.278	0.001
	None	523 (79.7)	354 (91.9)	169 (62.4)		
	Smaller	97 (14.8)	20 (5.2)	77 (28.4)		
	Larger	5 (0.8)	3 (0.8)	2 (0.7)		
	Completely	31 (4.7)	8 (2.1)	23 (8.5)		
**Childcare disagreement with children on consumption**					106.702	0.001
	None	475 (72.4)	337 (87.5)	138 (50.9)		
	Smaller	136 (20.7)	36 (9.4)	100 (36.9)		
	Larger	11 (1.7)	3 (0.8)	8 (3.0)		
	Completely	34 (5.2)	9 (2.3)	25 (9.2)		

*MOC, migrant older with children*.

### Social support and health status of the MOC

Table 3 shows the social support and health status of the MOC. Most of the MOC had a moderate level of social support (76.7%). More than half of participants had the same health status compared to peers (54.6%), and 67.5% of participants had the same health status compared to last year. A statistically significant difference between social support, health status compared to peers, and health status compared to last year, and unmet healthcare-seeking behavior was found based on the chi-squared test among the MOC in Jinan, China.

**Table 3:** Social support and health status of the MOC.

**Variables**		**Unmet healthcare-seeking behavior**			***χ^2^***	***P***
		**Total *n* (%)**	**No *n* (%)**	**Yes *n* (%)**		
SSRS	Mean (SD) = 39.46 ± 6.19					
					6.231	0.044
	Low ≤ 22	4 (0.6)	3 (0.8)	1 (0.4)		
	Moderate ≥23 and ≤44	503 (76.7)	282 (73.2)	221 (81.5)		
	High ≥45 and ≤66	149 (22.7)	100 (26.0)	49 (18.1)		
Health status compared to peers					14.273	0.006
	Much worse	16 (2.4)	11 (68.8)	5 (31.3)		
	Worse	68 (10.4)	32 (47.1)	36 (52.9)		
	Same	358 (54.6)	210 (58.7)	148 (41.3)		
	Any better	151 (23.0)	103 (68.2)	48 (31.8)		
	Much better	63 (9.6)	29 (46.0)	34 (54.0)		
Health status compared to last year					12.027	0.002
	Get better	88 (13.4)	46 (52.3)	42 (47.7)		
	Same	443 (67.5)	280 (63.2)	163 (36.8)		
	Get worse	125 (19.1)	59 (47.2)	66 (52.8)		

*MOC, migrant older with children; SSRS, Social Support Rating Scale*.

### The relationship between demographic characteristics, childcare disagreement with children, social support, health status, and unmet healthcare-seeking behavior

Table 4 shows the relationship between demographic characteristics, childcare disagreement with children, social support, health status, and unmet healthcare-seeking behavior using logistic regression. The Bonferroni correction results showed that two variables (source of living expenses and will of migration) were statistically significant, implying that the robustness check was passed and that the results of the binary logistic regression in this study were acceptable.

**Table 4:** The binomial logistic regression of demographic characteristics, disagreements, social support, health status, and unmet healthcare-seeking behavior of the MOC.

**Variables**	**Model 1**			**Model 2**			**Model 3**			**Model 4**		
	***P***	**OR**	**95%CI**	***P***	**OR**	**95%CI**	***P***	**OR**	**95%CI**	***P***	**OR**	**95%CI**
**Gender**												
Male		Ref			Ref			Ref			Ref	
Female	0.002	0.554	0.383–0.802	0.014	0.599	0.399–0.901	0.013	0.593	0.393–0.895	0.009	0.570	0.375–0.867
**Source of living expenses**												
Pension or salary		Ref			Ref			Ref			Ref	
Savings	0.567	1.248	0.585–2.661	0.457	1.375	0.594–3.180	0.488	1.349	0.579–3.145	0.494	1.345	0.575–3.148
Spouse's income	0.017	2.526	1.180–5.406	0.056	2.284	0.980–5.326	0.069	2.196	0.940–5.132	0.053	2.362	0.987–5.650
Child support	0.002	2.086	1.311–3.320	0.074	1.6	0.956–2.679	0.071	1.61	0.961–2.697	0.041	1.734	1.023–2.939
Government subsidy	0.002	4.199	1.688–10.446	0.002	5.33	1.822–15.791	0.002	5.566	1.883–16.453	0.003	5.339	1.796–15.874
**Monthly income**												
CNY 0–100 (USD 0–15.5)		Ref			Ref			Ref			Ref	
CNY 101–600 (USD 15.6–92.7)	0.010	0.383	0.185–0.795	0.096	0.497	0.218–1.132	0.095	0.494	0.216–1.130	0.117	0.510	0.220–1.183
CNY 601–2,000 (USD 92.8–309.2)	0.206	0.630	0.308–1.290	0.287	0.652	0.297–1.433	0.263	0.637	0.289–1.404	0.362	0.687	0.306–1.540
CNY ≥ 2,001 (USD ≥ 309.2)	0.678	0.826	0.335–2.036	0.871	1.087	0.399–2.961	0.939	0.961	0.349–2.648	0.929	0.954	0.341–2.673
**Household monthly income**												
CNY 0–100 (USD 0–15.5)		Ref			Ref			Ref			Ref	
CNY 101–1,000 (USD 15.6–154.5)	0.282	1.534	0.704–3.345	0.370	1.489	0.624–3.553	0.342	1.528	0.638–3.660	0.350	1.529	0.628–3.725
CNY 1,001–2,500 (USD 154.7–386.3)	0.566	1.267	0.565–2.838	0.777	1.137	0.469–2.756	0.757	1.151	0.473–2.799	0.593	1.280	0.517–3.168
CNY ≥ 2,501 (USD ≥ 386.4)	0.023	2.804	1.151–6.831	0.143	2.071	0.782–5.486	0.125	2.147	0.809–5.697	0.090	2.363	0.875–6.381
**Will of migration**												
Definitely will		Ref			Ref			Ref			Ref	
Definitely not	0.375	0.594	0.189–1.874	0.584	0.692	0.186–2.578	0.605	0.707	0.190–2.627	0.827	0.862	0.226–3.282
Partially not	0.303	0.526	0.155–1.784	0.342	0.524	0.138–1.992	0.346	0.520	0.133–2.029	0.330	0.508	0.130–1.984
Generally	0.042	0.464	0.221–0.972	0.184	0.565	0.243–1.312	0.235	0.594	0.252–1.402	0.243	0.589	0.243–1.432
Partially will	0.001	0.281	0.187–0.423	0.001	0.336	0.215–0.527	0.001	0.358	0.227–0.562	0.001	0.367	0.232–0.581
**Hire a nanny**												
No		Ref			Ref			Ref			Ref	
Yes	0.039	0.096	0.010–0.890	0.022	0.068	0.007–0.680	0.023	0.063	0.006–0.678	0.031	0.064	0.005–0.783
**Childcare disagreement with children on diet**												
None					Ref			Ref			Ref	
Smaller				0.279	1.577	0.692–3.594	0.251	1.628	0.709–3.742	0.314	1.539	0.665–3.562
Larger				0.987	0.979	0.075–12.779	0.951	0.922	0.071–11.932	0.869	0.811	0.067–9.804
Completely				0.515	2.256	0.195–26.086	0.609	1.914	0.159–23.027	0.560	2.127	0.168–26.917
**Childcare disagreement with children on dressing**												
None					Ref			Ref			Ref	
Smaller				0.015	0.345	0.147–0.811	0.012	0.332	0.140–0.785	0.024	0.366	0.153–0.877
Larger				0.315	0.270	0.021–3.466	0.352	0.297	0.023–3.826	0.312	0.262	0.020–3.510
Completely				0.309	4.751	0.236–95.823	0.396	3.753	0.177–79.443	0.321	5.031	0.207–122.510
**Childcare disagreement with children on education**												
None					Ref			Ref			Ref	
Smaller				0.436	1.398	0.601–3.250	0.311	1.555	0.662–3.651	0.262	1.646	0.689–3.933
Larger				0.131	0.146	0.012–1.777	0.180	0.171	0.013–2.258	0.190	0.186	0.015–2.303
Completely				0.328	2.915	0.342–24.829	0.255	3.568	0.398–31.961	0.279	3.537	0.359–34.811
**Childcare disagreement with children on consumption**												
None					Ref			Ref			Ref	
Smaller				0.006	0.414	0.221–0.774	0.010	0.435	0.232–0.817	0.009	0.430	0.227–0.813
Larger				0.045	0.176	0.032–0.962	0.071	0.209	0.038–1.146	0.100	0.233	0.041–1.325
Completely				0.297	0.202	0.010–4.081	0.402	0.272	0.013–5.735	0.358	0.223	0.009–5.443
**SSRS**												
High ≥45 and ≤66								Ref			Ref	
Low ≤ 22							0.927	1.124	0.092–13.661	0.926	1.129	0.087–14.592
Moderate ≥23 and ≤44							0.016	0.540	0.327–0.893	0.030	0.567	0.339–0.946
**Health status compared to last year**												
Get worse											Ref	
Same										0.198	1.595	0.784–3.245
Get better										0.031	1.869	1.057–3.306
**Health status compared to peers**												
Much better											Ref	
Much worse										0.176	2.879	0.621–13.340
Worse										0.327	1.586	0.631–3.988
Same										0.200	1.560	0.790–3.081
Better										0.058	2.044	0.976–4.281

MOC, migrant older with children; CNY, Chinese Yuan; SSRS, social support rating scale; OR, odds ratio; CI, confidence interval.

In Model 1, basic demographic information variables were included. The results showed that gender, source of living expenses, monthly income, household monthly income, will of migration, hiring a nanny, and unmet healthcare-seeking behavior among the MOC were statistically significant. When childcare disagreement with children variables was entered into Model 2, gender, source of living expenses, will of migration, and hiring a nanny remained significant, but the significance of monthly income and household monthly income disappeared. Childcare disagreement with children on dressing and childcare disagreement with children on consumption were also significant. In Model 3, social support was also significant.

In Model 4, female MOC were less likely to experience unmet healthcare-seeking behavior than men (OR = 0.570, 95%CI = 0.375–0.867, *p* = 0.009). The MOC with partial will of migration were less inclined to have unmet healthcare-seeking behavior than those with definite will of migration (OR = 0.367, 95%CI = 0.232–0.581, *p* < 0.001). The MOC who hired a nanny were less likely to report unmet healthcare-seeking behavior than those who did not have a nanny (OR = 0.064, 95%CI = 0.005–0.783, *p* = 0.031). The MOC who had smaller childcare disagreement with children on dressing were less likely to have unmet healthcare-seeking behavior than those who had none (OR = 0.366, 95%CI = 0.153–0.877, *p* = 0.024). The MOC who had smaller childcare disagreement with children on consumption were less inclined to have unmet healthcare-seeking behavior than those who had none (OR = 0.430, 95%CI = 0.227–0.813, *p* = 0.009). The MOC having moderate social support were less inclined to have unmet healthcare-seeking behavior than those having higher social support (OR = 0.567, 95%CI = 0.339–0.946, *p* = 0.030). The MOC whose health status compared to last year get better were more likely to have unmet healthcare-seeking behavior than those who did worse (OR = 1.869, 95% CI = 1.057–3.306, *p* = 0.031).

## Discussion

### Association between demographic characteristics and unmet healthcare-seeking behavior

The results of this study showed that 41.3% (*n* = 271) of the MOC experienced unmet healthcare-seeking behavior. This result was consistent with previous studies, which showed that migrants had a poor initiative in seeking treatment and were reluctant to seek medical care (36). The choice of healthcare-seeking behavior reflected the weak health awareness of the older migrant. Moreover, statistically significant correlations between gender, source of living expenses, will of migration, hiring a nanny, and unmet healthcare-seeking behavior were found in this study. Concerning gender, the results in Table 4 in this study showed that the MOC with women were less likely to report unmet healthcare-seeking behavior, this was similar with the study conducted by Zeng et al. who also found that female older individuals were more likely to utilize healthcare services (37). This may be due to women's relatively higher cautious health beliefs. Another study found that older men were significantly more likely to have inpatient disease compared to women, but there were no significant gender differences in decisions regarding hospitalization (38), which was different from our result. This might be due to the different research participants between our study and that study.

The results of this study showed that the MOC with source of living expenses from child support and a government subsidy were more likely to report unmet healthcare-seeking behavior than those with a pension or salary. This result was similar to a study conducted among the Karnataka older, which found that lack of earning capacity and financial dependence on others may cause them to forego treatment (39). The MOC with lower migration will (named as “partial will of migration” in Table 4) were less inclined to have unmet healthcare-seeking behavior than those with higher migration will (named as “definite will of migration” in Table 4). The result was different from Long's study, which found a positive association between willingness for long stay and the use of essential public health services (40). This might be because MOC with high migration may ignore or sacrifice their own healthcare service needs to provide better care for their younger generations (41). The MOC who hired a nanny were less likely to report unmet healthcare-seeking behavior than those did not have a nanny. This might be because when their grandchildren could be taken care by the nanny, it was more realizable and convenient for the MOC to seek healthcare services without disturbing their children's routine work and life arrangement.

### Association between childcare disagreement with children and unmet healthcare-seeking behavior

The MOC who had smaller childcare disagreement with children on dressing were less likely to have unmet healthcare-seeking behavior than those who had none, while the MOC who had smaller childcare disagreement with children on consumption were also found to be less likely to have unmet healthcare-seeking behavior than those who had none. The above results differed from the study by Lu and Deng, which found no statistically significant effect of intergenerational economic support of children on older adults' healthcare-seeking behavior (42). With the fast social and economic development, more and more differences in values and behaviors appeared between parents and children. According to Jorgensen et al.'s study, intergenerational upward social mobility may lead to a stressful relation between adult offspring and their older parents (43). Taking into account that 87.5% of the MOC were from the rural area (as shown in Table 1), most of the children of MOC should have been firstly born in the rural area and are currently working in the big cities. This indicated an intergenerational upward social mobility between the MOC and their children, but also brought a negative impact on the intergenerational relationship between them, such as the different understandings of dressing and consumption of the baby in this study. Wu and Chiou's study showed that poor intergenerational relationships were associated with a worse mental state among the elderly (44). The worse mental status of the MOC furtherly made them to seek healthcare services, in other words, the MOC who had smaller childcare disagreement with children were less likely to have unmet healthcare-seeking behavior than those who had none.

### Association between social support and unmet healthcare-seeking behavior

In this study, a significant association between social support and unmet healthcare-seeking behavior was observed, specifically, the current results demonstrated that participants with a moderate level of social support were less likely to experience unmet healthcare-seeking behavior than those with a high level. This was different from a previous study, which showed that low social support was associated with refraining from seeking medical care and medical non-adherence (45). Long's study showed that outer social support is positively associated with the use of health services in older migrants in China (46). Another research demonstrated that, compared to the medium- or low-level social support group, seafarers with a high level of social support had better scores in the general facet of health, quality of life, physical health, psychological health, social relations, and environment domains (33). Social support networks play a significant role in promoting healthy aging (47). High levels of social support were more beneficial to an individual's physical and mental health than lower levels of support among older adults (48). This may indicate that the MOC with a high level of social support were in good health and had a lower likelihood of healthcare-seeking behavior, while those with a moderate level of social support needed more healthcare-seeking behavior, that is, the MOC with a moderate level of social support were less likely to experience unmet healthcare-seeking behavior than those with a high level.

### Association between health status and unmet healthcare-seeking behavior

The MOC whose health status compared to last year get better were more likely to have unmet healthcare-seeking behavior than those who worsened. The result was the same as the study by Shao, which found that migrants with moderate and severe status of symptoms have a higher likelihood of seeking healthcare services (49). This result was also the same as the study that showed people who thought their illness was serious were more likely to visit a doctor (50). This was different from a previous study, which showed that old-aged migrants in better health condition had a significantly sufficient utilization rate (51). It was important for the MOC to pay attention to their own health conditions and enhance their health status.

### Implications

In view of these findings, the following policy recommendations were given. Firstly, the community could develop recreational programs to reinforce the relationship between the neighbors and friends of the MOC to increase their social support network. Secondly, low-educated and low-income MOC deserve more focus, the government should pay more attention to increase accessibility to healthcare services and medical subsidies to achieve health equality (52). Thirdly, the primary healthcare center is advised to conduct more health education on MOC, which could increase the awareness of the healthy lifestyle to promote their own health status and improve their healthcare-seeking behavior (53). Finally, children of internal or cross-nation older migrants should spend more time with family members and be filial to their parents to reduce the contradiction and create a harmonious family atmosphere, which was also the basic requirement of filial piety in traditional Confucianism Chinese culture (54).

### Limitations

This study has some limitations. Firstly, individual health levels in this study were based on a self-report measure, which might have a certain impact on the results of our data analysis. Compared with a comprehensive health evaluation scale, a self-rated assessment of health is a subjective evaluation, which may be less objective and lead to recall bias. Secondly, the total number of family members was not collected in this study, and the household monthly income per capita could not be calculated. Thirdly, the use of variables in data analysis may need more consideration because continuous variable may be more acceptable in regression analysis rather than the categorical variable. Fourthly, this study was a cross-sectional design, so causality cannot be determined.

## Conclusions

In this study, 41.3% of the MOC were found to have unmet healthcare-seeking behavior. The MOC whose health status compared to last year get better were more likely to have unmet healthcareseeking behavior; while who were women, had partial will of migration, hired a nanny, had smaller childcare disagreement with children on dressing, had smaller childcare disagreement with children on consumption, and had moderate social support, were less likely to experience unmet healthcare-seeking behavior. In summary, smaller childcare disagreement with children, moderate social support, and worse health status would result in less unmet healthcare-seeking behavior of the MOC. As an initial study on the effects of childcare disagreement with children, social support, and health status on unmet healthcare-seeking behavior among the MOC in Jinan, China, the results of this study provide evidence-based information for the government and the public on enhancing the healthcare-seeking behavior of the MOC.

## Data availability statement

The raw data supporting the conclusions of this article will be made available by the authors, without undue reservation.

## Ethics statement

Ethical review and approval was not required for the study on human participants in accordance with the local legislation and institutional requirements. Written informed consent from the participants was not required to participate in this study in accordance with the national legislation and the institutional requirements.

## Author contributions

XS analyzed the data and drafted this manuscript. FK applied the fund to support this study, designed this study, completed the questionnaire design, supervised and combined the collected data, instructed the writing, performed statistical analysis and data processing, and provided comments on the modification of this manuscript. SL provided some valuable comments on the draft to further polish it. All authors read and approved the final manuscript. All authors read and agreed to the published version of this manuscript.

## Funding

This study was supported and funded by the National Natural Science Foundation of China (No. 71804094), China Postdoctoral Science Foundation (No. 2016M592161), Natural Science Foundation of Shandong province (No. ZR2016GB02), Postdoctoral Science Foundation of Shandong province (No. 201603021), and The Fundamental Research Funds of Shandong University (Nos. 2015HW002 and 2018JC055).

## Conflict of interest

The authors declare that the research was conducted in the absence of any commercial or financial relationships that could be construed as a potential conflict of interest.

## Publisher's note

All claims expressed in this article are solely those of the authors and do not necessarily represent those of their affiliated organizations, or those of the publisher, the editors and the reviewers. Any product that may be evaluated in this article, or claim that may be made by its manufacturer, is not guaranteed or endorsed by the publisher.
